# Case Report: Epidermodysplasia verruciformis misdiagnosed as pityriasis alba in a child: a diagnostic pitfall in facial hypopigmented lesions

**DOI:** 10.3389/fimmu.2026.1851554

**Published:** 2026-06-29

**Authors:** Siyi Pan, Zhengwei Tan, Yanan Zhang

**Affiliations:** 1 Dermatology Department, Wenzhou Hospital of Integrated Traditional Chinese and Western Medicine, Wenzhou, China; 2 Department of Hematology, The First Affiliated Hospital of Zhejiang Chinese Medical University (Zhejiang Provincial Hospital of Chinese Medicine), Hangzhou, China

**Keywords:** children, dermatoscopy, differential diagnosis, epidermodysplasia verruciformis, hypopigmented macules, misdiagnosis, pityriasis alba

## Abstract

**Objective:**

Epidermodysplasia verruciformis (EV) is a rare genodermatosis with susceptibility to specific HPVs and risk of malignant transformation. Atypical EV presenting as isolated facial hypopigmented macules closely mimicking pityriasis alba can lead to misdiagnosis. This case report aims to clarify key differential diagnostic points through a misdiagnosed pediatric case.

**Methods:**

Clinical data of an 11-year-old girl with EV, initially misdiagnosed as pityriasis alba and confirmed by dermoscopy and histopathology, were retrospectively analyzed. Clinical, dermoscopic, and histopathological distinctions were identified, and early diagnosis and treatment strategies were discussed.

**Results:**

The patient presented with a 6-month history of asymptomatic, progressively enlarging hypopigmented macules on the forehead. Prior treatment for pityriasis alba was ineffective. Dermoscopy revealed irregular pale white papular structures on a pale yellowish-brown background without scale. Histopathology showed characteristic gray-blue, vacuolated epidermal cells, confirming EV. Treatment with topical 5% imiquimod and cryotherapy led to partial regression at 12 weeks, with no new lesions or adverse effects.

**Conclusion:**

Atypical EV with isolated facial hypopigmented macules in children is easily misdiagnosed as pityriasis alba. For refractory, progressively enlarging lesions, dermoscopic screening, with histopathological confirmation reserved for equivocal cases, facilitates early diagnosis. The three-tier diagnostic system integrating clinical, dermoscopic, and histopathological evaluation improves early diagnostic accuracy and reduces long-term malignant risk.

## Introduction

Epidermodysplasia verruciformis (EV) is a rare hereditary dermatosis whose pathogenesis is closely linked to host innate immunodeficiency resulting from loss-of-function mutations in the TMC6 (EVER1) and TMC8 (EVER2) genes on chromosome 17q25. These genes encode transmembrane proteins that regulate keratinocyte antiviral immunity, and the resulting immune defect leads to persistent infection with β-genus human papillomavirus (HPV) (1). The majority of cases of this disease occur in childhood or adolescence. The typical clinical manifestations are generalized flat, wart-like papules and tinea versicolor-like pigmentary patches. In severe or longstanding cases, EV-associated lesions may undergo malignant transformation, most commonly progressing to cutaneous squamous cell carcinoma (cSCC), which represents the most serious life-threatening complication of this disease. Classical EV manifests as generalized flat-wart-like papules and seborrheic keratosis-like plaques, with high clinical recognizability. In contrast, atypical tinea versicolor-like EV presents solely as isolated or scattered hypo- or hyperpigmented macules without overt verrucous hyperplasia. Its predilection for sun-exposed sites, including the pediatric face, and a clinical phenotype that closely overlaps with pityriasis alba render it a high-risk subtype for clinical misdiagnosis and underdiagnosis.

Currently, the majority of reported cases of EV in the literature focus on patients with typical skin lesions, combined malignant transformation, or a clear family history. Research on EV in children with normal immune function, no family history, and isolated facial hypopigmented macules as the only manifestation, systematically comparing the differential diagnosis with pityriasis alba, is extremely scarce. In clinical practice, such cases are readily diagnosed as pityriasis alba, leading to missed diagnosis of EV, causing affected children to miss opportunities for early intervention and increasing their long-term risk of malignant skin transformation.

Herein, we report a case of EV in an 11-year-old girl presenting with a solitary facial hypopigmented macule, who was initially misdiagnosed as pityriasis alba at an external hospital. We systematically established a key differential diagnostic framework for distinguishing this atypical EV subtype from pityriasis alba in children. We discussed the value of dermoscopy in the non-invasive screening of pediatric facial hypopigmented lesions. This case report aims to provide practical diagnostic references for primary care clinicians, enhance their ability to recognize early disease, and reduce misdiagnosis and missed diagnosis of EV.

## Case presentation

An 11-year-old girl presented to the dermatology outpatient department of our hospital with a complaint of “white macules on the forehead for more than 6 months, with progressive increase for 1 month”. The patient first developed scattered pale white macules on the forehead without any obvious precipitating factor 6 months previously, without any subjective symptoms such as itching, pain, or burning sensation, and with no previous history of trauma or inflammation. She was treated at a local community hospital, diagnosed with “pityriasis alba”, and treated with topical medical moisturizing emollients and intermittent low-potency glucocorticoid cream for 2 months. The skin lesions did not improve, and their number progressively increased during the preceding month, with partial fusion; therefore, she presented to our hospital.

The patient had no previous history of serious diseases, no history of primary immunodeficiency, no long-term use of glucocorticoids or immunosuppressants, and no history of blood transfusion or close contact with infectious diseases. There was no family history of similar skin diseases or hereditary skin diseases. The patient had normal development, and no abnormalities were found on systemic physical examination.

Specialist physical examination: Multiple scattered hypopigmented macules, slightly elevated above the skin surface, were seen on the forehead, with varying sizes ranging from approximately 1 mm to 5 mm in diameter. Some lesions were arranged in a linear pattern and had fused into irregular patches with indistinct boundaries. The surface of the lesions was smooth, without obvious scales, scabs, exudation, erosion, or atrophy. The morphology and color of local eyebrows and forehead vellus hair were normal, and no similar lesions were found on the skin and mucosa of other parts of the body.

**Figure 1:**
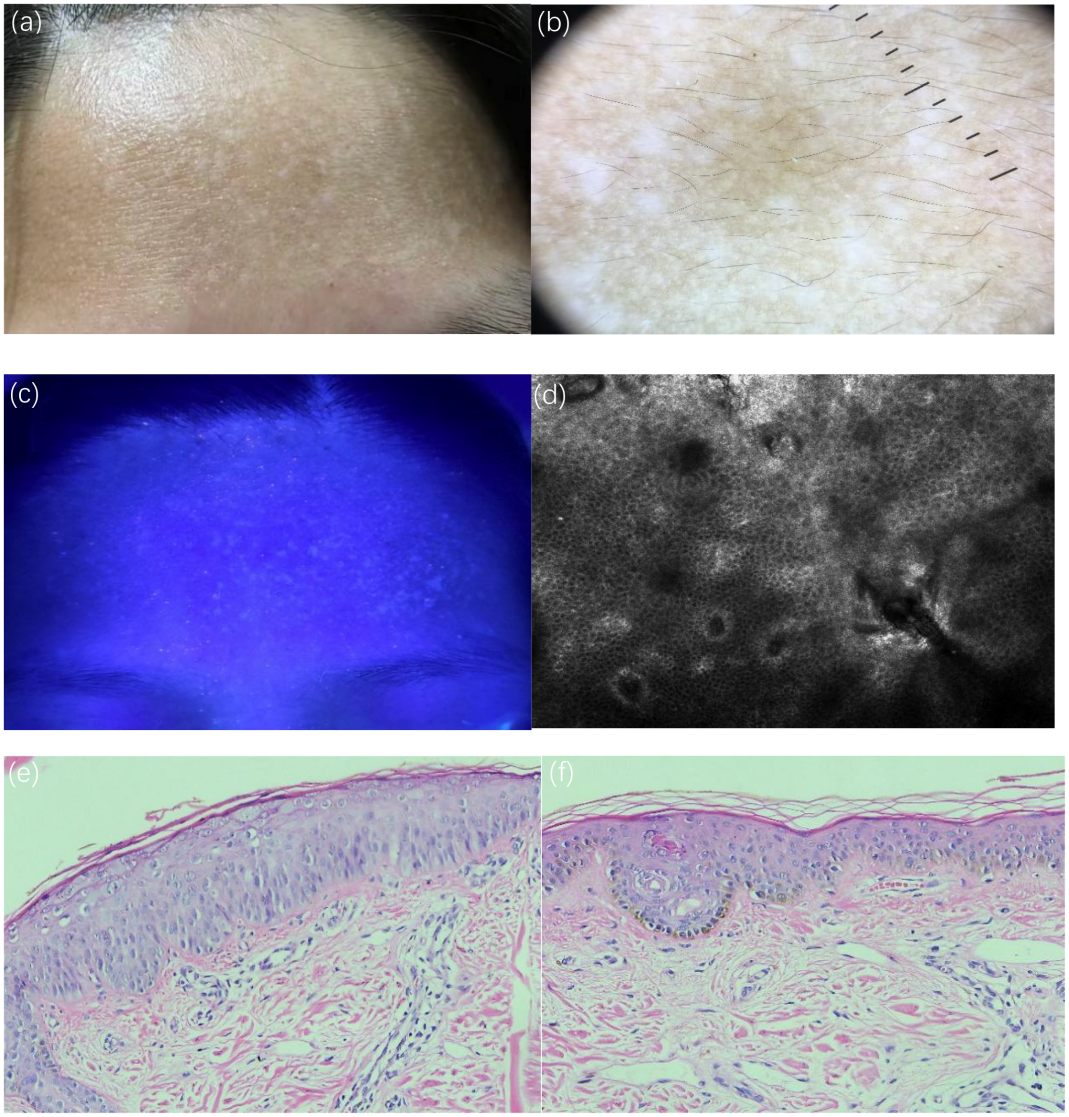
**(A)** Multiple scattered hypopigmented macules, slightly elevated above the skin surface, were observed on the forehead. **(B)** Dermoscopy revealed an uneven, mottled, light yellowish-brown background with multiple 1–6 mm pale white hypopigmented macules, characterized by pathognomonic feathered/halo-like margins and extensive coalescence into geographic/reticular patches. **(C)** The lesions showed pale blue-white fluorescence. **(D)** The normal, regular honeycomb pattern of the spinogranular layer was disrupted, appearing disorganized and irregular. **(E, F)** Histopathological examination staining demonstrated diffuse koilocytosis in the mid-upper epidermis, accompanied by mild acanthosis and superficial perivascular lymphocytic infiltrate, consistent with epidermodysplasia verruciformis. The stratum corneum showed mild basket-weave hyperkeratosis without significant parakeratosis.

Auxiliary examination (**Figure 1**): (1) Dermatoscopic examination: Against a pale yellowish-brown skin background, multiple pale white flat papular structures of different sizes and irregular shapes were observed, with unclear lesion boundaries and a tendency toward partial fusion. No scales, abnormal vascular structures, follicular plugs, or abnormal pigment structures were found on the surface of the lesions. (2) Wood’s lamp examination: The lesions showed pale blue-white fluorescence, with no bright blue-white complete depigmentation, and no yellow-white fungal fluorescence. (3) Fungal microscopy: No Malassezia spores or hyphae were identified in lesional scales. (4) Histopathological examination: The epidermis showed hyperkeratosis and mild acanthosis, with scattered and focally distributed characteristic gray-blue vacuolated cells throughout the epidermis. These cells exhibited enlarged cell volume, pale cytoplasm, and perinuclear vacuolation. A small amount of lymphocyte-based inflammatory cell infiltration was seen in the superficial dermis; no melanocyte loss or atypical changes were found. (5)Reflectance confocal microscopy: The regular honeycomb pattern of the normal spinogranular layer of the epidermis was disrupted, presenting a disorganized and irregular honeycomb-like architecture, indicating abnormal arrangement of epidermal keratinocytes and supporting HPV-induced cytomorphological changes.

Diagnosis and treatment: Based on the patient’s clinical history, specialist physical examination, dermatoscopic features, and histopathological findings, the final diagnosis was epidermodysplasia verruciformis. The treatment plan consisted of topical application of 5% imiquimod cream to the lesions every other night. The patient was instructed to adhere to daily sun protection measures to avoid excessive ultraviolet exposure, take medication regularly, return to the clinic every 4 weeks to evaluate changes in the lesions, and undergo long-term follow-up to monitor the lesions’ malignant potential.

After treatment with topical 5% imiquimod cream every other night combined with strict sun protection, no new lesions developed during the follow-up period. At 4 weeks of treatment, the color of the lesions began to deepen; at 8 weeks, the area of individual lesions had decreased by approximately 30%; and at 12 weeks, partial regression of lesions was observed, with an overall lesion reduction rate of approximately 50%. No local irritation, erythema, or other adverse effects were observed. The patient was followed up for 6 months, and the condition remained stable, with no recurrence or signs of malignant transformation.

## Discussion

### Clinical phenotypic heterogeneity of pediatric EV and the root cause of misdiagnosis

This case represents an atypical tinea versicolor-like EV, the most misdiagnosed subtype, with clinical features distinct from classical EV. Classical EV comprises three types, namely, flat-wart, tinea versicolor-like, and seborrheic keratosis-like lesions, among which the flat-wart type is the most common (2). The tinea versicolor-like type presents as hypo- or hyperpigmented patches without verrucous hyperplasia and is frequently underrecognized (2). Our patient had disease onset in childhood, no family history, normal immune function, and a solitary hypopigmented macule on the forehead as the only manifestation, without generalized lesions; these features constituted the primary reason for the initial misdiagnosis. Genetic testing of TMC6/EVER1 and TMC8/EVER2 is the gold standard for EV diagnosis and should be prioritized in suspected cases. When encountering asymptomatic pale patches on a child’s face, primary care clinicians often rely on routine diagnostic patterns and diagnose pityriasis alba, overlooking rare diseases such as EV, which led to the misdiagnosis in this case. The absence of generalized lesions, verrucous hyperplasia, and a family history further complicated the clinical diagnosis and represents a major challenge in the current recognition of atypical EV.

### Genotype–histopathology–phenotype correlation in EV

Loss-of-function mutations in TMC6/EVER1 or TMC8/EVER2 impair keratinocyte antiviral immunity, enabling persistent β-HPV infection and inducing characteristic vacuolar degeneration of keratinocytes (histologically manifested as gray-blue vacuolated cells) (3). These cellular changes further disrupt cutaneous pigment metabolism and epidermal architecture, manifesting clinically as hypopigmented macules and dermoscopically as pathognomonic feathered margins and reticular confluence. The present case fully demonstrates this correlation; a definitive diagnosis can be established based on clinical, dermoscopic, and histopathologic findings, even in the absence of genetic testing.

### Core differential diagnostic system between EV and pityriasis alba

A three-tier diagnostic protocol consisting of clinical screening, dermoscopic triage, and histopathological confirmation for selected cases provides a structured approach to reducing misdiagnosis of EV in children with facial hypopigmented macules. Clinically, EV should be suspected over pityriasis alba when smooth, non-scaly, progressively expanding lesions on sun-exposed sites do not respond to moisturization or low-potency corticosteroids, whereas pityriasis alba typically presents with fine scaling, an atopic background, a self-limiting course, and a favorable response to emollients (4).

On dermoscopy, pityriasis alba shows ill-defined hypopigmentation with perifollicular sparing and fine scale, without specific vascular or pigment structures (4). In contrast, this EV case exhibited a mottled pale yellowish-brown background with multiple irregular pale macules, feathered/halo-like borders, and geographic/reticular confluence, features attributable to HPV-induced keratinocyte vacuolation and clearly distinct from pityriasis alba. This dermoscopic pattern provides a non-invasive marker that improves differentiation, particularly for isolated pediatric facial EV, for which data remain scarce (5).

Histopathologically, EV is confirmed by the presence of epidermal vacuolated keratinocytes, whereas pityriasis alba shows only mild parakeratosis, spongiosis, and basal hypomelanosis, without vacuolated cells (6, 7). Key differential points are summarized in **Table 1**.

**Table 1:** Three-tier differential diagnostic criteria for pityriasis alba versus early hypopigmented epidermodysplasia verruciformis.

Differential dimension	Pityriasis alba	Early hypopigmented epidermodysplasia verruciformis
Clinical feature differentiation	•Predominantly distributed on the cheeks • Surface with fine bran-like scales	• Distributed on sun-exposed areas • Smooth surface without scales
Dermoscopic feature differentiation	• Ill-defined hypopigmented areas • Characteristic perifollicular pigment sparing • Fine superficial scaling	• Pathognomonic ill-defined feathered/halo-like borders • Partial coalescence into geographic and reticular patches
Histopathological differentiation	• Mild spongiosis and decreased basal melanin	• **Diffuse vacuolated keratinocytes (koilocytes) in the mid- to upper epidermis**

1. This table is specifically designed for early hypopigmented EV (the most easily misdiagnosed subtype), as advanced EV with verrucous hyperplasia has distinct clinical features that rarely cause diagnostic confusion. 2. The bolded items represent the **core diagnostic markers** with the highest differential value.

### Early intervention and long-term management of pediatric EV

There is currently no curative treatment for EV. Early diagnosis, timely intervention, and lifelong follow-up are the core principles for improving the long-term prognosis of children (2). For localized EV lesions in children, topical immunomodulators (e.g., 5% imiquimod cream) combined with physical therapy (such as liquid nitrogen cryotherapy or laser therapy) constitutes the first-line treatment. Imiquimod can clear HPV-infected keratinocytes and inhibit viral replication by activating local innate immunity, thereby effectively controlling lesion progression and preventing the development of new lesions. For generalized lesions, systemic retinoic acid drugs can be used; however, their adverse effects in pediatric patients require careful monitoring.

The core of long-term management in pediatric patients with EV is the prevention of malignant transformation. Clinical data show that the malignant transformation rate of skin lesions in EV patients can reach 30%–60%, with the majority of cases occurring in patients with a disease course of more than 20 years. Ultraviolet irradiation is the primary risk factor for malignant transformation of lesions. This case indicates that, for children with localized atypical EV, topical 5% imiquimod cream alone can effectively control lesion progression and promote partial regression while avoiding scarring and pigmentary abnormalities that may be induced by physical therapy, thus representing a preferred therapeutic regimen for pediatric patients. The patient in this case was diagnosed in childhood and is expected to have a long disease course; therefore, lifelong strict sun protection and regular follow-up every 3 to 6 months are recommended to comprehensively evaluate lesion progression. If the lesions demonstrate rapid enlargement, ulceration, bleeding, or other manifestations, histopathological examination should be performed immediately to exclude malignant lesions.

### Limitations

This report has several limitations. Genetic analysis of *TMC6/EVER1* and *TMC8/EVER2* was not performed due to financial constraints, precluding definitive molecular confirmation. Genetic testing remains the diagnostic gold standard and should be pursued whenever feasible. Future large-scale studies are needed to characterize genetic variants of atypical pediatric EV and their phenotype correlations.

## Conclusion

Atypical EV presenting solely as isolated facial hypopigmented macules in children shares highly overlapping clinical features with common pityriasis alba, making it a high-risk subtype for misdiagnosis and missed diagnosis. The three-tier differential diagnostic workflow established in this case report effectively addresses this clinical challenge. For lesions refractory to conventional moisturizing and corticosteroid therapy and showing progressive enlargement, timely non-invasive dermoscopic screening, followed by histopathological examination when indicated, enables early diagnosis and intervention in EV. Early diagnosis and standardized management of childhood EV can effectively control disease progression, facilitate the development of lifelong follow-up and malignant transformation prevention strategies, and significantly improve patients’ long-term prognosis.

## Data Availability

The original contributions presented in the study are included in the article/supplementary material. Further inquiries can be directed to the corresponding author.
